# Decreased oxygen saturation levels in neonates with transposition of great arteries: Impact on appearance of cerebral veins in susceptibility-weighted imaging

**DOI:** 10.1038/s41598-017-15591-3

**Published:** 2017-11-13

**Authors:** Rajeev Kumar Verma, Desislava Keller, Sebastian Grunt, Sandra Bigi, Christian Weisstanner, Roland Wiest, Jan Gralla, Damian Hutter, Bendicht Wagner

**Affiliations:** 1 University Institute of Diagnostic and Interventional Neuroradiology, Inselspital, University of Bern, Bern, Switzerland; 2 Institute of Radiology and Neuroradiology, Tiefenau Hospital, Division Stadt, Inselgroup, Bern, Switzerland; 3 University Department of Pediatrics, Division of Pediatric Neurology, Development and Rehabilitation, Inselspital, University of Bern, Bern, Switzerland; 4 University Department of Pediatrics, Division of Pediatric Cardiology, Inselspital, University of Bern, Bern, Switzerland; 5 University Department of Pediatrics, Division of Pediatric Intensive Care Medicine, Inselspital Bern, University of Bern, Bern, Switzerland

## Abstract

Purpose of this study was to investigate a potential correlation between the pattern of cerebral veins (CV) on susceptibility-weighted imaging (SWI) and blood oxygen saturation, as well as preoperative brain injury, in neonates with transposition of the great arteries (TGA). Eleven neonates with TGA underwent MRI preoperatively, including SWI, T1- and T2-weighted scans. Images were retrospectively evaluated and appearance of CV was graded from 0 (normal appearance) to 3 (severe prominent appearance). White matter injuries (WMI) and strokes were analysed. Results were correlated with preductal arterial oxygen saturation. As findings one subject showed a normal CV appearance (grade 0) whereas 10 showed pathological prominent CV (grades 1–3); median 2. Mean oxygen saturation ranged between 67.5% and 89.0% (median 81.0%). CV grade and mean oxygen saturation correlated significantly (p = 0.011). WMI were absent in 5 cases, mild in 4, and moderate in 2 cases. We conclude, that SWI has the potential to be used to estimate the current hypoxic burden on brain tissue in TGA newborns by assessing the prominence of the CV.

## Introduction

Severe congenital heart disease (CHD) is a common cause of childhood morbidity with a prevalence of 8 per 1000 live births in Europe^1^. Advances in diagnostics, cardiac surgery and intensive care medicine have significantly reduced mortality by enabling early surgical repair of most severe defects^2,3^. In neonates with transposition of the great arteries (TGA), the neonatal arterial switch operation for correction is widely performed because it has good cardiovascular and survival outcomes. However, neurodevelopmental disabilities have emerged as the most common long-term complication of CHD in the surviving subjects^4,5^. Neurocognitive impairment affects many functions, e.g. executive function, motor development, memory and attention^6,7^. More than 50% of neonates with CHD show neurological abnormalities preoperatively^8^. Conventional MRI studies showed that brain injury with white matter lesions and cerebral strokes occurred before surgery in newborns with CHD^9–14^. A possible cause is preoperative hypoxia^15–17^. Studies using advanced MRI techniques revealed further abnormalities, e.g. reduced or diminished white matter microstructure in adolescents with CHD, using diffusion tensor imaging (DTI)^18^, or elevated lactate in neonates with CHD, using magnetic resonance spectroscopic imaging (MRSI)^19^. Susceptibility-weighted imaging (SWI) is a relatively new, but already well-established sequence. It is a high-resolution gradient-echo (GRE) MRI sequence with fully velocity-compensated pulse. Differences in the susceptibility properties of various tissues can be differentiated by post-processing the magnitude images using a phase mask, thereby emphasizing the magnetic properties of different susceptibility effects. SWI has improved diagnostic accuracy, e.g. through detecting and evaluating intracranial haemorrhage, haemorrhagic transformation of stroke or calcification^20–22^. In the cerebral veins (CV), decreased oxygen saturation correlates with an increase of de-oxygenated haemoglobin that leads to a prominent appearance of these veins^23^. This phenomenon is used for diagnostic purposes. Prominent CV were described in stroke patients in the perfusion-disturbed areas after thromboembolic occlusion^20–24^ and in patients with hemiplegic migraine^25^ due to hypoperfusion. The opposite effect, with diminished CV, was demonstrated in patients with hyperperfusion in status epilepticus and in hyperventilated patients^26,27^. A correlation between blood oxygen levels and prominence of CV has been also described^27,28^. Changes in blood oxygenation levels are well-known to also affect the signal intensity of cerebral veins in SWI^29^. Kitamura *et al*. introduced at first a scoring system of “prominence of vein” using SWI to asses neonatal hypoxic – ischemic injury. In this study a correlation of patients with “abnormal” “prominence of vein” and abnormal intermediate-term to long-term neurologic outcome was found^30^. Neonates with TGA suffer from low arterial oxygen saturation. It is assumed that the degree and duration of preoperative hypoxia is a risk factor for later neurocognitive deficits^8^. While white matter lesions in MRI scans reveal chronic brain injury, the prominence of CV in the SWI is a dynamic feature of the current shortage of oxygen supply.

Given these points, the aim of this study was to investigate whether a presumed general prominence of CV could be used diagnostically to estimate the current hypoxic burden on brain tissue. We hypothesized a correlation between the preoperative oxygen saturation in patients suffering from TGA and the prominence of CV in SWI, which reflects the deoxygenated share of haemoglobin. Further, a correlation between preoperative brain injury (amount of white matter lesions) and the grading of prominence of CV was analysed.

## Materials and Methods

### Patient data

The retrospective study was approved by the local Ethics Committee (Kantonale Ethikkommission Bern, Switzerland) and was carried out according to the Declaration of Helsinki^31^. An informed consent of the patients was not necessary due to the retrospective study design.

Subjects who met the inclusion criteria for this descriptive case study were term neonates with confirmed TGA who underwent routine preoperative MRI, and were hospitalized in our paediatric intensive care unit. Only neonates with TGA were included, because of the quite similar circulation anomaly in these patients, but variable blood oxygen saturation. During the MR examination all patients were sedated with either midazolam i.v. (total dose between 0.1 mg and 3.1 mg) and/or chloral hydrate p.o. (total dose between 60 mg and 300 mg). In individual cases rocuronium i.v. and/or ketamine i.v. was injected (up to 3.2 mg, and up to 3.5 mg, respectively). None of the children received propofol medication, because of the risk for propofol infusion syndrome. Mandatory sequences for this study were SWI, T1- and T2-weighted images. A further inclusion criterion was availability of values for arterial oxygen saturation (measured by means of both arterial blood gas analysis and pulse oximetry) and arterial carbon dioxide (CO_2_) (measured by means of arterial blood gas analysis) that had been recorded on the same day that the patient underwent MRI. Concentrations of serum glucose in mmol/L, blood haemoglobin in g/L, blood pH, and heart rate in beats/min were noted. Further, for a higher objectivity three values of preductal peripheral oxygen saturation (SaO_2_) during or close to MRI examination were determined: first the blood arterial oxygen saturation (blood gas analysis data closest to SWI acquisition time), second the single peripheral oxygen saturation measurement (pulse oximetry data closest to SWI acquisition time), and third the mean of peripheral oxygen saturation measurements, measured in between 2 h before and after SWI acquisition (pulse oximetry data). Patient data were excluded if image quality was poor, e.g. due to motion artifacts, if MRI data had not been acquired due to cardiovascular instability, or if the patient had a complex form of TGA, e.g. TGA with a single ventricle.

### Data acquisition

All imaging studies were performed using 1.5 T Siemens scanners (Magnetom Avanto and Magnetom Aera; Siemens Medical Solutions, Erlangen, Germany) with 12-channel head coils. The MRI protocol included axial SWI, T1 turbo spin echo (TSE) and T2 TSE. The following parameters were used.

#### Magnetom Avanto

SWI parameters: TR 49 ms, TE 40 ms, FoV read 230 mm, FoV phase 68.8%, voxel size 0.9 mm × 0.7 mm × 1.2 mm, slice thickness 1.2 mm, acquisition time 5:07 min. The SWI and minimum intensity projection (mIP) images were generated automatically by the scanner software. T1 TSE parameters: TR 1000 ms, TE 13 ms, FoV 200 × 200, slice thickness 3 mm, acquisition time 4:51 min. T2 TSE parameters: TR 4260 ms, TE 113 ms, FoV 200 × 200, slice thickness 3 mm, acquisition time 4:43 min. Diffusion-weighted imaging (DWI) parameters: TR 6400 ms, TE 89 ms, FoV 230 × 230, slice thickness 3 mm, acquisition time: 2:54 min.

#### Magnetom Aera

SWI parameters:TR 49 ms, TE 40 ms, FoV read 230 mm, FoV phase 90.6%, voxel size 0.9 mm × 0.9 mm × 2.0 mm, slice thickness 2.0 mm, acquisition time 3:27 min. The SWI and mIP images were generated automatically by the scanner software. T1 TSE parameters: TR 1380 ms, TE 12 ms, FoV 200 × 200, slice thickness 3 mm, acquisition time 4:47 min. T2 TSE parameters: TR 4380 ms, TE 107 ms, FoV 200 × 200, slice thickness 3 mm, acquisition time 2:50 min. DWI parameters: TR 5700 ms, TE 64 ms, FoV 230 × 230, slice thickness 4 mm, acquisition time: 3:31 min.

### Data analysis

**Figure 1:**
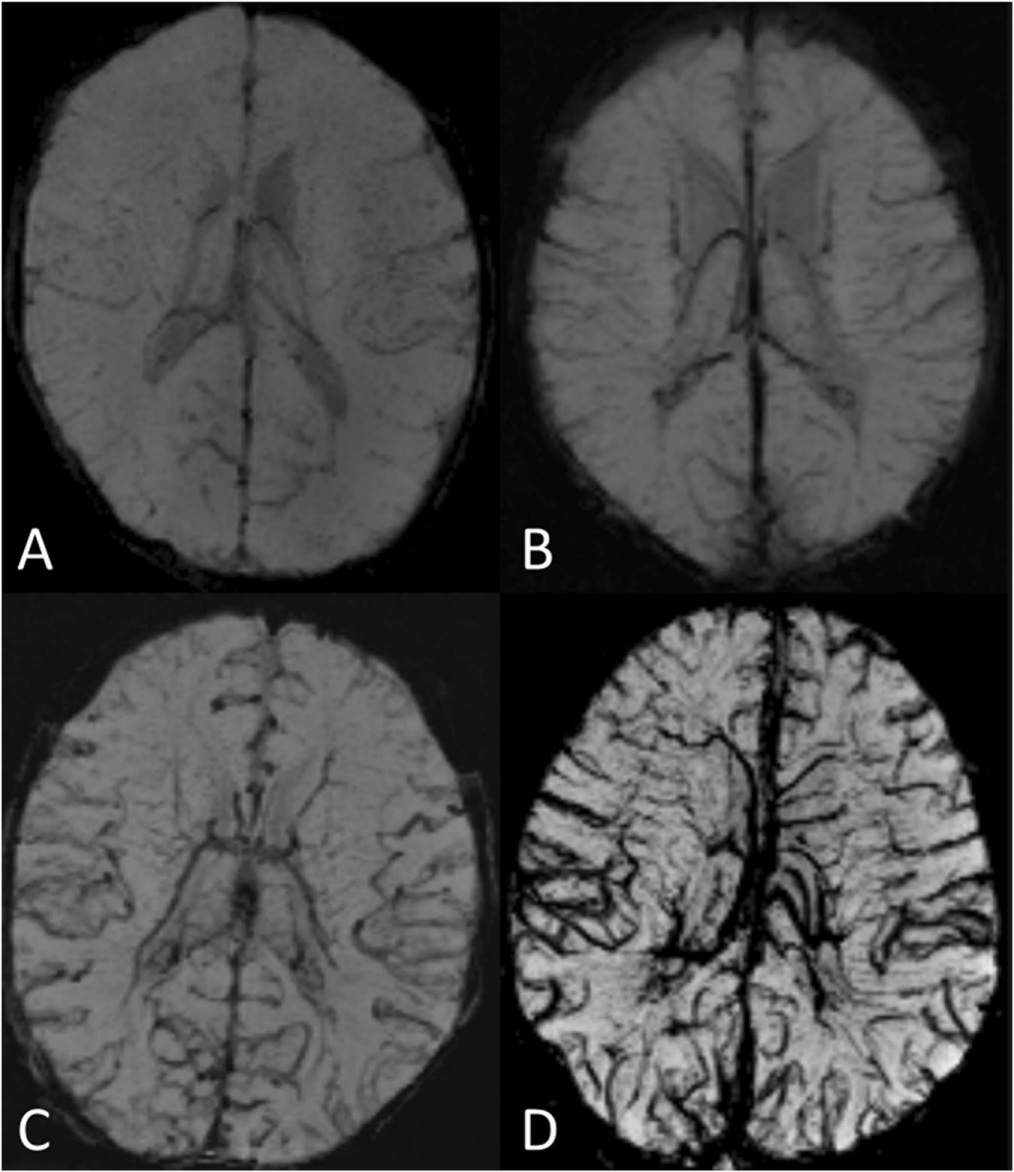
SWI images in minimum intensity projection (miP) as examples of grading of cerebral veins. (**A**) Grade O: normal appearance of cerebral veins (images of a 2-day-old healthy subject with normal art. oxygen saturation of 95%); (**B**) Grade 1: mildly prominent deeper veins and cortical veins (patient No. 5). (**C**) Grade 2: moderately prominent cortical and deeper veins (patient No. 8). (**D**) Grade 3: distinctly prominent cortical and deeper veins, prominent veins in the semioval centre bilateral (patient No. 1).

MRI sequences for all patients were reviewed independently by two board-certified neuroradiologists (RKV and DK for SWI, T1-weighted and T2-weighted images; RKV and CW for DWI). Other than knowing that these were TGA cases, they were blinded to the clinical course. MRI scans were assessed on our picture archiving and communication system (PACS) separately, in a standardized order: first SWI, followed by T1-weighted and T2-weighted images. When examining the SWI, both neuroradiologists classified the global prominence of CV according to 4 grades in the minimum intensity projection images (mIP): Grade 0: normal signal intensity, number, and diameter of cortical veins and deep veins (one or more veins up to 0.9 mm and 1.1 mm in diameter, respectively); no visible veins in the semioval centre. Grade I: slightly decreased signal intensity, increased number and diameter of cortical veins and deep veins (one or more veins up to 1.1 mm and 1.3 mm in diameter, respectively); virtually no visible veins in the semioval centre. Grade II: moderately decreased signal intensity, increased number and diameter of cortical veins and deep veins (one or more veins up to 1.4 mm and 1.7 mm in diameter, respectively); slightly to moderately visible veins in the semioval centre. Grade III: distinctly decreased signal intensity, increased number and diameter of hypointense cortical veins and deep veins (one or more veins up to 1.7 mm and 2.4 mm in diameter, respectively); distinctly visible veins in the semioval centre (see Fig. 1). This grading might vary depending on individual anatomical conditions, the strength of the magnetic field or the MR manufacturer, and therefore be imprecize. Hence, we included further typical characteristics of prominent CVA (increased number of cerebral veins, increased signal intensity of cerebral veins, increased diameter of cerebral veins, and visible veins in the semioval centre) for a more objective and differentiated grading. Further, for two reasons we did not exactly use the “prominence of vein” scoring system introduced by Kitamura *et al*.^30^, but modified it. For evaluation only the deep medullary veins were used in this system. Meanwhile many studies have revealed the usefulness of both cortical and deep veins for evaluation of vein prominence^22–25^. For a higher sophistication we included these veins in our scoring system. Secondly, in some recently published studies diminished cerebral veins occur in case of a higher cerebral blood flow or increased oxygen supply most likely resulting in higher oxygenated haemoglobin levels^26–28^. Since TGA patients suffer from low oxygen saturation levels, leading to increased deoxygenated haemoglobin levels, we have not applied Grade 1 of the Kitamura scoring (absent deep medullary veins).

**Figure 2:**
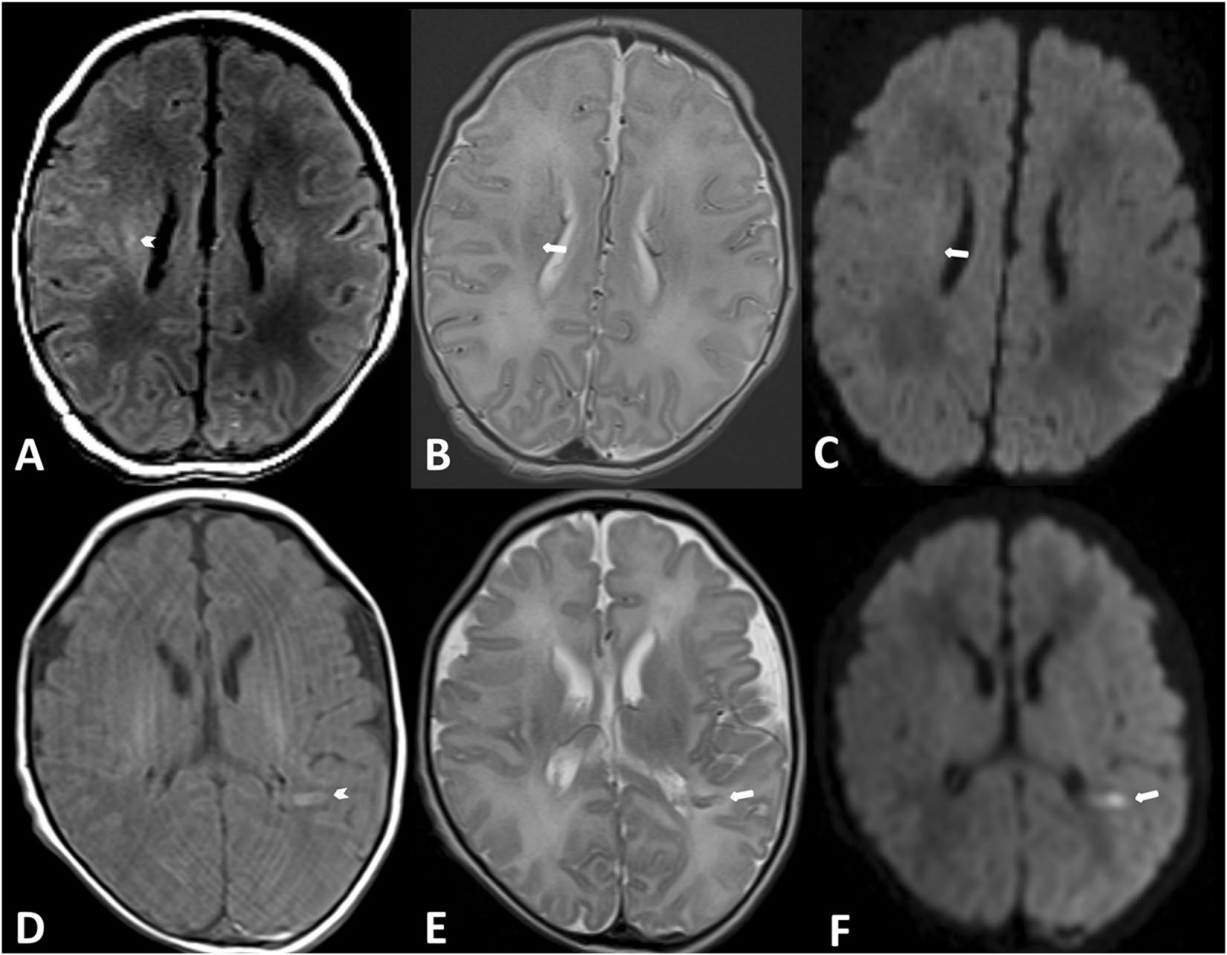
Examples of a WMI (upper row) and a stroke (lower row). A WMI is seen in the right semioval centre with T1w hyperintense signal intensity and no signal changes in T2w and DWI (patient No. 8). An acute stroke is seen in the white matter on the left, with signal changes in T1w and T2w and diffusion restriction in the DWI (patient No. 3).

For evaluation of white matter injuries (WMI) and cerebral strokes we used a score previously described by McQuillen *et al*.^13^. In T2- and T1-weighted images, WMI and white matter strokes were analysed and counted: WMI were defined as lesions measuring <2 mm of abnormal T1-hyperintensity or of low intensity on T1-weighted images in the absence of marked T2-hypointensity^13,16^. Severity of WMI was classified as absent (0 areas of T1 signal abnormality); mild (≤3 areas of T1 signal abnormality measuring <2 mm); moderate (>3 areas of T1 signal abnormality measuring <2 mm or areas of T1 signal abnormality measuring >2 mm but covering <5% of the cerebral hemisphere); or severe (T1 signal abnormality covering >5% of the cerebral hemisphere) (see Fig. 2A to C). Cerebral strokes were defined as focal regions of ≥3 mm with or without diffusion restriction on DWI^12^ (see Fig. 2D to F). In a final session, a consensus reading was performed in cases of disagreement.

### Statistical analysis

For statistical analysis the SPSS statistics software package was used (IBM, Armonk, N.Y., USA). A non-parametric, two-sided Kendall rank correlation coefficient test was applied to verify whether the two variables (prominence of CV and arterial blood oxygen levels) were statistically dependent. A p-value of <0.05 was considered significant. Median and total range for all parameters was determined (SWI grade, WMI, white matter strokes, arterial blood oxygen saturation, arterial blood pCO_2_, blood glucose, blood haemoglobin, blood pH, single monitored oxygen saturation measurement, and mean monitored oxygen saturation value).

For interrater reliability of findings in SWI, T1w and T2w sequences in MRI scans, a Cohens-Kappa test was performed and the interrater reliability was classified according to Landis and Koch^32^.

### Data availability

The datasets generated during and/or analysed during the current study are not publicly available due to preclusion from dissemination following Swiss Federal Law Regulations, but are available from the corresponding author on reasonable request.

## Results

### Subjects and clinical data

Between March 2011 and July 2015, 11 patients (8 male and 3 female; mean age 5.7 days at time of MRI acquisition) fulfilled the inclusion criteria.

Arterial blood oxygen saturation ranged between 66.0 and 88.0% (median 75%; n = 11) and was low in all subjects (normal range 93–98%). Monitored preductal single oxygen saturation value closest to the time of SWI acquisition ranged between 64.0% and 86.0% (median 84.5; n = 10), and monitored mean preductal oxygen saturation values (measured in between 2 h before and after SWI acquisition) ranged between 68.0% and 89.0% (median 82.0%; n = 10).

Arterial pCO_2_ ranged between 36 and 55 mm Hg (median 41 mm Hg; n = 11). Blood glucose ranged between 4.5 and 6.3 mmol/L, median 5.2 (n = 10). Haemoglobin values were between 124 and 177 g/L, median 144 (n = 11). pH ranged between 7.34 and 7.45, median 7.37 (n = 11); see Table 1 for details.

**Table 1:** PFO, persisting foramen ovale; PDA, patent ductus arteriosus; ASD, atrial septum defect; VSD, ventricular septum defect; n.a., not available. Values of arterial O2-saturation in %, arterial pCO2 in mmHg, Glc in mmol/L, and Hb in g/L were taken from blood. Monitored SaO2 measurements (single measurement closest to SWI acquisition, and mean +/−2 hours of SWI acquisition) were pulse oximetry data. HR, heart rate in beats per minute.

Patient No.	Age(sex)	Diagnosis	SWI grade	WM injuries (total areas)	WM stroke (total areas)	Acute ischemic lesions (total areas)	blood art. O2-saturation (%)	blood art. pCO2 mmHg	Glc mmol/L	Hb g/L	pH (normal range	time between blood- taking and MRI acquisition	closest single SaO2 measurement to SWI acquisition (in %) monitoring data (time distance in parentheses)	SaO2 mean (in %) monitoring data (no. of measurements in brackets)	Heart rate
1	8d (m)	d-TGA	3	1	0	1	66	39	4.9	171	7.45	7 h 57 min	64 (5 min)	70.8 (5)	156
2	3d (m)	d-TGA; low cardiac output syndrome	1	1	0	0	75	41	4.5	137	7.34	3 h 55 min	84 (1h18min)	83 (2)	128
3	7d (m)	d-TGA with restrictive PFO and restrictive PDA	1	10	3 (MCA left)	2	83	36	5.1	153	7.38	8 h 50 min	86 (15 min)	89 (3)	144
4	9d (m)	d-TGA with restrictive ASD	2	1	0	0	71.5	43	5.8	124	7.38	2 h 17 min	75 (30 min)	73.8 (5)	160
5	6d (m)	TGA with interrupted aortic arch type A and distinct VSD, restrictive PFO, and restrictive PDA	1	0	0	1	83	45	6.3	140	7.36	1 h 54 min	85 (15 min)	85.7 (3)	160
6	2d (m)	d-TGA with distinct ASD and PDA	3	0	0	0	75	38	5.2	148	7.37	1 h 36 min	86 (10 min)	75.3 (3)	164
7	13d (m)	d-TGA with restrictive ASD	2	1	0	1	66.7	41	5.7	134	7.37	1 h 22 min	64 (10 min)	67.5 (4)	130
8	4d (f)	d-TGA with VSD, valvular and subvalvular pulmonary stenosis	2	4	0	0	71.7	55	4.7	177	7.35	2 h 58 min	n.a.	n.a.	n.a.
9	4d (f)	d-TGA	2	0	0	0	82	37	5	144	7.45	8 h 47 min	72 (20 min)	79 (4)	144
10	5d (f)	d-TGA, ASD, VSD, aortic coarctation, and low output syndrome	0	0	0	0	88	42	5.4	159	7.42	7 h 42 min	85 (40 min)	86 (2)	182
11	2d (m)	d-TGA, ASD, VSD, aortic coarctation, and low output syndrome	1	0	0	0	78	43	n.a.	142	7.34	1 h 52 min	85 (10 min)	84.5 (2)	150

Blood CO_2_ and blood haemoglobin were in the upper standard value range in 2 subjects. Blood glucose was normal in all patients.

### Imaging findings

One subject showed a normal cerebral veins appearance (CVA) (grade 0) while 10 showed prominent CVA (4 patients with grade 1, 4 patients with grade 2, and 2 patients with grade 3); range between grade 0 and 3; median 2. Severity of WMI was graded as absent in 5 cases with no WMI, mild in 4, and moderate in 2 cases. Only one patient showed white matter strokes in the territory of the left middle cerebral artery with 3 lesions >2 mm. Furthermore, in one patient two acute ischaemic areas were found, in 3 patients 1 acute ischaemic area was found and 7 patients had no acute ischaemia (Table 1).

**Figure 3:**
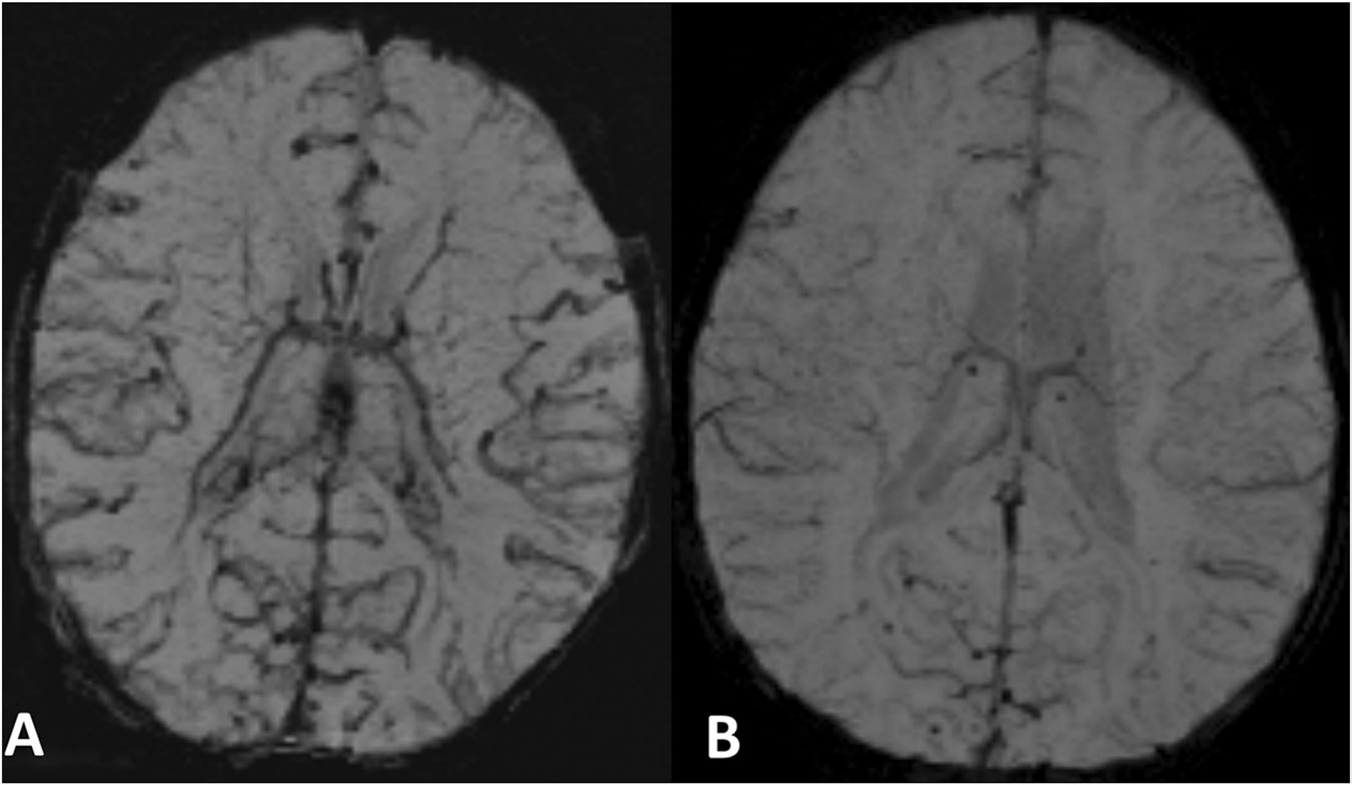
SWI images of a patient (No. 8) as an example before (Fig. 3A) and after surgery (Fig. 3B). After surgery the CV appearance improved distinctly becoming normal. Before surgery oxygen saturation was 72% (CV appearance Grade II), after surgery oxygen saturation was 95% (CV appearance Grade 0).

Patient No. 8 underwent follow-up MR imaging postoperatively, with an oxygen saturation of 95% and a normalized CV appearance Grade of 0 (see Fig. 3B).

### Statistical analysis

The interrater reliability was high (Kappa = 0.869 [p < 0.001]) for the grade of cerebral venous prominence in SWI, as well as for the WMI in T1-weighted and T2-weighted images (Kappa = 0.861 [p < 0.001]). Interrater reliability for acute ischaemic findings was high too (Kappa = 0.907 [p = 0.02]).

**Figure 4:**
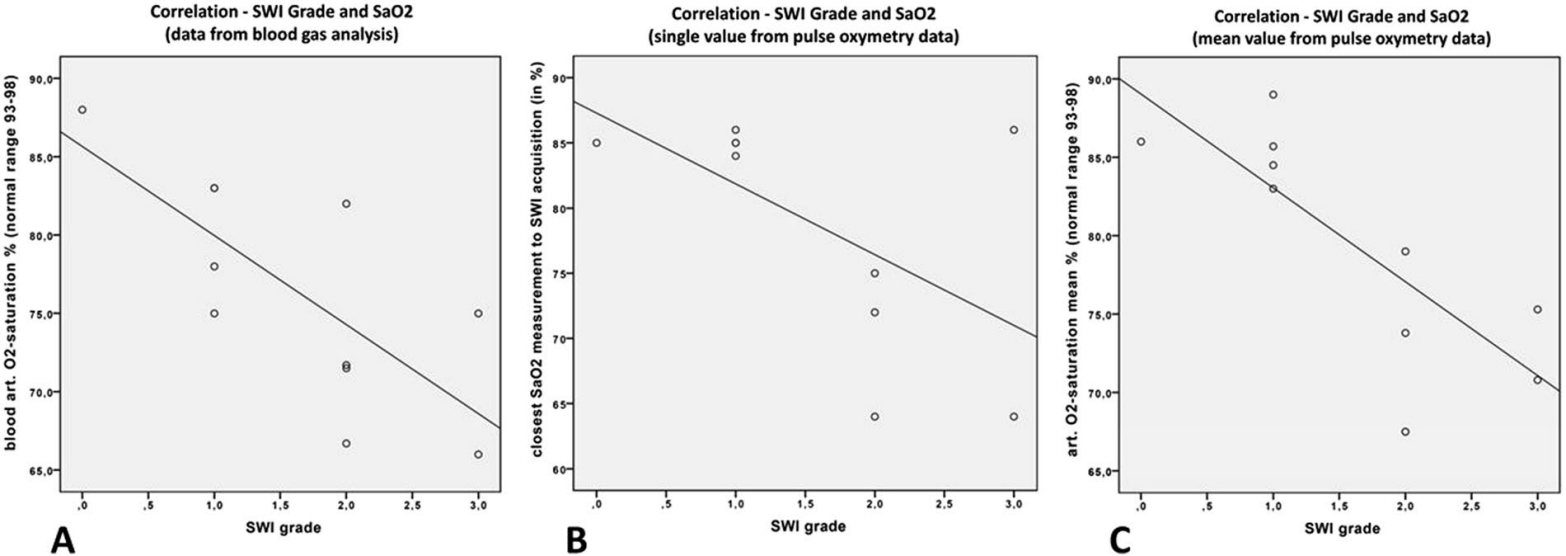
Correlation between oxygen saturation and grade for appearance of cerebral veins in SWI (scatter plots for illustration). For correlation analysis between oxygen saturation and grade for appearance of cerebral veins in SWI, a non-parametric, two-sided Kendall rank correlation coefficient test was used (Fig. 4A–C: scatter plots for illustration with corresponding regression lines). Figure 4A demonstrates a significant correlation between SWI grade and SaO2 of arterial blood in % (p = 0.01, Kendall’s tau = −0.657; regression line y = 85.66–5.69 * x). Figure 4B shows no significant correlation between SWI grade and SaO2 in % of single peripheral pulse oximetry data, because of an outlier: Pt. No. 6 with SWI grade 3 and 86% SaO2 (p = 0.147; Kendall’s tau = −0.401; regression line y = 87.29–5.43 * x). Figure 4B reveals a significant correlation between SWI grade and SaO2 in % of mean peripheral pulse oximetry data (p = 0.011; Kendall’s tau = −0.680; regression line 89.03–5.98 * x).

Three values of oxygen saturation were analysed (Fig. 4 for illustration): the blood arterial oxygen saturation (Fig. 4A, blood data closest to SWI acquisition time), single peripheral oxygen saturation measurement (Fig. 4B; pulse oximetry data closest to SWI acquisition time), and mean of peripheral oxygen saturation measurements, measured ± 2 h of SWI acquisition (Fig. 4C; pulse oximetry data). A significant correlation was found for blood arterial oxygen saturation (p = 0.01, Kendall’s tau = −0.657; Fig. 4A), and for mean peripheral oxygen saturation (p = 0.011, Kendall’s tau = −0.680; Fig. 4C), but not for single peripheral oxygen saturation, measurement closest to SWI measurement (p = 0.147, Kendall’s tau = −0.401; Fig. 4B) due to a statistical outlier (patient No. 6: SWI grade 3 and 86% SaO2).

No significant correlation was seen between severity of WMI and prominence of CV in SWI (p = 0.721,Kendall’s tau = 0.099), nor was any significant correlation found between acute ischaemia in DWI and prominence of CV in SWI (p = 1.0, Kendall’s tau = 0.0) or between CVA and blood CO_2_, blood glucose, blood haemoglobin, and blood pH (p-values between 0.243 and 0.679, Kendall’s tau between −0.302 and 0.194).

## Discussion

The main finding of our investigation is a significant correlation between the prominence of CVA and blood oxygen saturation levels. In our study only one newborn showed a normal appearance of the CV, whereas 10 revealed a pathological venous pattern with presumed higher deoxyhaemoglobin levels, significantly correlating with the arterial blood oxygen saturation (blood arterial oxygen saturation and monitored mean oxygen saturation). Only monitored single oxygen saturation measurements showed no significant correlation, because of an outlier value (patient No. 6 with 86% oxygen saturation and prominence of CV of grade 3). To the best of our knowledge this is the first report showing a significant correlation between blood arterial oxygen saturation levels and the extent of prominent CV in SWI of newborns suffering from TGA. However, in a recently published study by Öztoprak on 19 adult patients with pulmonary embolism, a significant correlation between presence of prominent veins on SWI and hypoxemia^33^ was found, which is in line with our results. Further, our findings are in accordance with a recently published experimental study by Patzig *et al*.^34^. In their study 16 healthy adult volunteers underwent MRI under room air conditions, short-term hypoxia (7 minutes before and during the MRI scan), and long-term hypoxia (8.5 hours before and during the MRI scan). The 9 subjects who were finally included in the study showed significantly lower signal intensities of CV in the scans under conditions of hypoxia. However, our study is the first to investigate a patient population with chronically low arterial blood oxygen levels.

Blood CO_2_ is another parameter with an influence on CVA. For example, in a recently published study, a correlation between hyperventilation (in which CO_2_ is expelled leading to a vasoconstriction with lower cerebral blood flow) and higher signal intensity in CV was demonstrated, indicating a lower venous blood oxygenation level during hyperventilation^28^. Sedlacik *et al*. revealed a significant correlation between high etCO_2_ and cerebral blood flow with weak venous contrast in SWI^35^ in spontaneously breathing patients sedated with propofol. Since etCO_2_ and blood bicarbonate levels show a significant correlation^36^, blood CO_2_ might have influenced the CVA in our study. The difference between etCO_2_ and PaCO_2_ is stated to be 2–5 mm Hg with higher values for PaCO_2_^37^. Based on the findings of Sedlacik *et al*. etCO_2_ should be kept as low as possible to obtain a good venous contrast in SWI. With a maximal etCO_2_ value of 30–35 mmHg CBF in sedated children is not artificially elevated^38,39^. Although in our subjects, blood CO_2_ was normal, with a median of 41 mm Hg it might have influenced the CVA, even by subtracting 2–5 mm Hg, since it is still higher than 30–35 mm Hg and might lead to an increase of CBF. However, in case of an elevated blood CO_2_ and CBF a weak venous contrast would be expected. In our subjects a stronger venous contrast is observed, therefore we assume the influence of blood CO_2_ is presumably low to negligible. Further blood parameters with a potential to lead to bias were in the normal range (median of blood glucose 5.2 mmol/L; median of blood haemoglobin 144 g/L; and median pH of blood, 7.37). These findings indicate that the changes in CVA are most likely caused by the blood oxygen saturation levels, correlating with other investigations^28,29,33,34^.

Many studies have demonstrated that newborns with complex CHD have abnormal brain development with brain injuries and delayed brain maturation^9–11^. Preoperatively, neonates with TGA exhibit brain injury which is associated with hypoxia^15,17^. It can be assumed that if hypoxia improves in TGA patients, the neuroanatomical and developmental outcome improves too^40^. A technique currently used to reveal brain injury is MRI. Using diffusion-weighted T1w and T2w images, WMI and strokes can be detected. A correlation between WMI and neurological abnormalities has been described in neonates with severe CHD before surgery^16^. A study using magnetic resonance spectroscopy, which is an advanced neuroimaging technique, revealed an elevated lactate in neonates with TGA, indicating an abnormal brain metabolism^19^, even in subjects in whom MRI showed no evidence of preoperative brain injury. Diffusion tensor imaging studies revealed regions of reduced white matter^9,18^.

Our investigation showed no correlation of WMI, acute or chronic strokes, and SWI findings; e.g. patient No. 3 showed a clear discrepancy with 10 WMI lesions, but a mean oxygen saturation of 89% and a prominence of CV of grade 1. These results were expectable, since both the oxygen saturation measurement and the prominence of the CV provide a “snapshot” reflecting the current hypoxic burden, while WMI or strokes are unique events leading to chronic gliotic tissue damage. Nine of 11 subjects had no WMI, or only one lesion, but 10 of the 11 showed a pathological cerebral venous pattern.

The prominence of CVA in neonates with TGA in SWI is a new finding in this context, indirectly demonstrating an abnormal oxygen supply. Compared to chronic changes such as WMI, SWI has the advantage that it can demonstrate current acute changes in venous haemoglobin composition by CVA (see Fig. 3 as example). Therefore, SWI can potentially be used to estimate insufficient brain oxygen in neonates suffering from TGA, which might be important e.g. in postoperative control MRI to assess whether brain oxygen supply has improved. A relatively new method, for assessing cerebral oxygen saturation in neonates, is near-infrared spectroscopy (NIRS)^41^. Both techniques evaluate the “venous weighted” cerebral haemoglobin values. NIRS has some clear advantages. It evaluates the cerebral oxygen saturation, is non-invasive, measures cerebral oxygen saturation continuously, and is as such a valuable bedside tool. However, NIRS has some limitations including a limited penetration depth of the NIR light, i.e. reaching the cerebral cortex only, the instability of NIRS values, and the lack of a direct reference against which to correlate the values^42^. This might lead to a false higher cerebral oxygen saturation values in individual cases. Further, the derived NIRS-signal contains extracerebral “contamination” from bone and skin tissue. SWI has the ability to detect prominent cerebral veins in the whole brain in a standardized manner and therefore provides additional clinically meaningful information, despite the more work-intensive data acquisition. Since a brain MRI in children with congenital heart disease is carried out anyway, SWI in this context can be acquired quite easily.

Our study does however have limitations. Cerebral metabolic rate of oxygen and cerebral blood flow were not measured during MRI acquisition. Although proprofol was not applied to our patients, we cannot fully rule out an influence on cerebral metabolism and cerebral blood flow, caused by an anesthesic used in our patients, as described in previous studies for midazolam, chloral hydrate or ketamine^43–45^. Midazolam can slightly decrease cerebral blood flow^43^, which might contribute to the prominence of CV, because of higher deoxygenated hemoglobin levels. In contrast, ketamine might lead to a mild increase of cerebral blood flow with no effect on the metabolic rate^45^, which might contribute to lesser prominent CVs. For chloral hydrate both, a decrease of cerebral blood flow (resulting in higher deoxygenated hemoglobin levels) and a decrease of cerebral glucose utilization (resulting in higher oxygenated hemoglobin levels) was described^44^. Hence, an effect on the prominence of CV on SW images is more likely neutralized.

Further, using a retrospective study design we analysed a standard SWI sequence and graded the prominence of CV visually. Although the outcome was in line with our hypothesis, a more objective quantification of venous prominence would be helpful. An example of a relatively new approach is quantitative susceptibility mapping (QSM), using SWI for measuring oxygen saturation; it was introduced by Haacke *et al*.^46^ and has the potential to provide quantitative estimations of oxygen saturation in CV. This technique uses phase images and has been applied to CV in stroke patients for estimation of oxygen saturation in a recent study^47^. Both SWI and QSM are gaining increasing acceptance in clinical practice^48^. In future studies, acquisition of SWI with phase images in neonates with TGA would help to quantify the extent of oxygen shortage in CV.

Another limitation of the present study is the small number of patients. A larger cohort is needed to confirm our results.

In conclusion, the appearance of prominent cerebral veins in SWI is a pathological finding in 10 of 11 newborns with TGA in our study. It indicates an increase of deoxygenated hemoglobin, and thus lack of oxygenated hemoglobin. Our study demonstrated a significant correlation between the appearance of CV in SWI and the arterial blood oxygen levels in neonates with TGA. SWI has the potential to be used to estimate the current hypoxic burden on brain tissue by the effect on the appearance of CV. As a dynamic sequence SWI reflects the oxygen shortage in a “snapshot”, while other conventional sequences e.g. T1w or T2w images show chronic or subacute ischaemic changes. Therefore SWI might be a helpful additional sequence to the MRI protocol for TGA patients, to clarify the possibility and the extent of cerebral hypoxia (e.g before and after surgery), and rule out other intracranial pathologies, like hemorrhagic transformation of ischemic regions. Further studies with a larger cohort and SWI phase images would be beneficial to test our findings objectively by quantifying the cerebral venous oxygen saturation.
